# Role of Salicylic Acid and Components of the Phenylpropanoid Pathway in Basal and Cultivar-Related Resistance of Oilseed Rape (*Brassica napus*) to *Verticillium longisporum*

**DOI:** 10.3390/plants8110491

**Published:** 2019-11-11

**Authors:** Xiaorong Zheng, Birger Koopmann, Andreas von Tiedemann

**Affiliations:** Department of Crop Sciences, Section of Plant Pathology and Crop Protection, Georg August University, 37077 Göttingen, Germany; bkoopma@gwdg.de

**Keywords:** salicylic acid, phenolic acids, *NahG*, gene expression, enzymatic analysis

## Abstract

Enhanced resistance is a key strategy of controlling ‘Verticillium stem striping’ in *Brassica napus* caused by the soil-borne vascular pathogen *Verticillium longisporum*. The present study analyses the role of a broad range of components in the phenylpropanoid and salicylic acid (SA) pathways in basal and cultivar-related resistance of *B. napus* towards *V. longisporum*. A remarkable increase of susceptibility to *V. longisporum* in SA-deficient transgenic *NahG* plants indicated an essential role of SA in basal resistance of *B. napus* to *V. longisporum*. Accordingly, elevated SA levels were also found in a resistant and not in a susceptible cultivar during early asymptomatic stages of infection (7 dpi), which was associated with increased expression of *PR1* and *PR2*. In later symptomatic stages (14 or 21 dpi), SA responses did not differ anymore between cultivars varying in resistance. In parallel, starting at 7 dpi, an overall increase in phenylpropanoid syntheses developed in the resistant cultivar, including the activity of some key enzymes, phenylalanine ammonium lyase (PAL), cinnamyl alcohol dehydrogenase (CAD) and peroxidase (POX) and the expression of key genes, *PAL4*, *CCoAMT*, *CCR*, *POX*. As a consequence, a remarkable increase in the levels of phenolic acids (*t*-cinnamic acid, *p*-coumaric acid, caffeic acid, ferulic acid, sinapic acid) occurred associated with cultivar resistance. A principal component analysis including all 27 traits studied indicated that component 1 related to SA synthesis (*PR1*, *PR2*, *POX*, level of free SA) and component 2 related to lignin synthesis (level of free ferulic acid, free *p*-coumaric acid, conjugated *t*-cinnamic acid) were the strongest factors to determine cultivar-related resistance. This study provides evidence that both SA and phenolic acid synthesis are important in cultivar-related resistance, however, with differential roles during asymptomatic and symptomatic stages of infection.

## 1. Introduction

*Verticillium longisporum* (VL) is a soil-borne vascular fungal pathogen with host specificity to Brassicaceae [1]. The pathogen widely occurs in oilseed rape production regions in Europe and North America [2,3,4,5]. Oilseed rape (*Brassica napus*) is the most important crop for oil production in Europe and Canada and the prevalent host of *V. longisporum* [6]. Due to the relatively short crop rotation and increased area of oilseed rape cultivation, incidence of ‘Verticillium stem striping’ is on the rise and threatens oilseed rape production. In the absence of their host, the melanized microsclerotia of *V. longisporum* remain dormant and viable in soil for several years [6,7,8]. As control of *V. longisporum* with fungicides has not been successful [9], the most promising measure against this disease is breeding for effective resistance.

Salicylic acid (SA) is an important phytohormone which has been shown to play a role not only in local resistance but also in the activation of systemic acquired resistance (SAR) against pathogens [10]. SA can be synthesized either via the phenylalanine or isochorismate pathways in *Arabidopsis* [11,12]. Application of SA or its analogues (benzo (1,2,3)-thiadiazole-7-carbothioic acid S-methyl ester, BTH) enhances the resistance of plants against pathogens, while in SA-deficient *NahG* transformed plants the susceptibility to pathogens was increased [13,14]. However, surprisingly, in *B. napus,* a positive correlation between the concentration of SA in shoot extracts and the biomass of *V. longisporum* colonizing the plant was reported [15].

In *Arabidopsis*, soluble phenylpropanoids are involved in defense responses to *V. longisporum* [16]. In oilseed rape, genomic loci (QTL) for a number of phenylpropanoids were found to co-localize with the QTL for *V. longisporum* resistance [17]. Lignin, an important compound among phenylpropanoids, is mainly deposited in secondary cell walls, which may provide strength for preventing direct penetration by the fungus. Besides, several phenolic acids, intermediates in the lignin biosynthetic pathway, were accumulated in vascular tissues after infection of oilseed rape with *V. longisporum [18]*.

Although previous studies have indicated a role of SA and lignin biosynthesis in resistance responses of oilseed rape to *V. longisporum*, the role of specific components and the interplay between these pathways during infection is not yet understood. Therefore, the aim of this study was to explore the role in resistance of a larger set of metabolites, enzymes and genes involved in the biosynthesis of SA and lignin during infection of *B. napus* with *V. longisporum*. More specifically, we followed three hypotheses, (1) that SA is required in basal resistance of *B. napus* to *V. longisporum*, (2) that the phenylpropanoid pathways related to lignin synthesis determine cultivar-related resistance to *V. longisporum* and (3) that biosynthesis of SA and phenolic acids are in a competitive relationship. 

## 2. Results

### 2.1. V. longisporum Disease Development and Plant Colonization in NahG Transformed Oilseed Rape

**Figure 1:**
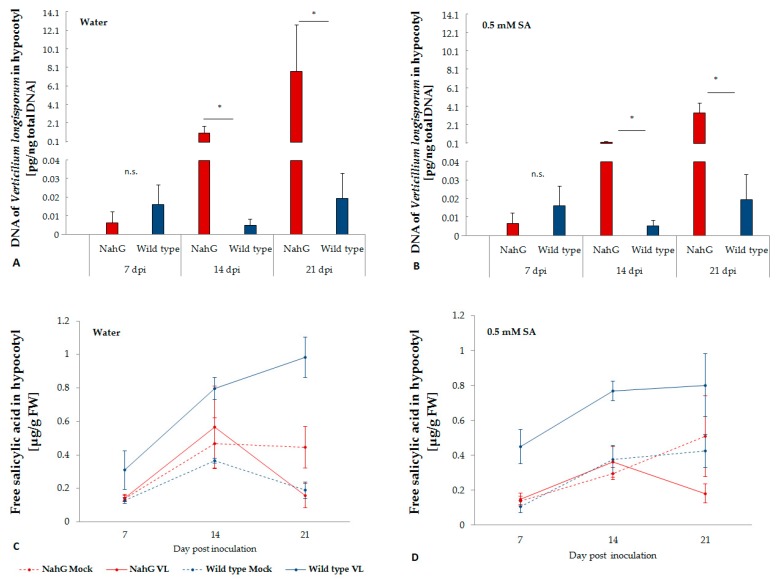
Colonization of in *B. napus* cv. Drakkar and its *NahG* transformant with *V. longisporum* with (**A**) or without (**B**) exogenous application of salicylic acid and the endogenous free salicylic acid content in the hypocotyl after infection (**C**,**D**). Bars indicate standard errors. Mean data were obtained from four biological replications. Asterisks on the bars indicate significant differences between two genotypes at the same time point (LSD, *p* < 0.05).

Typical symptoms of *V. longisporum* infection on *B. napus* under greenhouse or climate chamber conditions were leaf yellowing, vein blacking, premature senescence of leaves and stunting. In the *NahG* transformants, severe symptoms were observed already two weeks after inoculation, while in the wild type plants, only relative mild symptoms occurred at 21 dpi. In addition, diseased transformant plants showed a severely crippled, deformed shoot growth at 21 dpi. Except for the first week after inoculation, significantly higher levels of disease severity were recorded in *V. longisporum* infected *NahG* transformants than in the wild type plants. However, exogenously supplied SA by root dipping prior to inoculation did not effectively reduce disease severity (Table 1). The stunting effect of *V. longisporum* was significantly stronger on *NahG* transformants than on wild type plants. No significant effects by *V. longisporum* on the dry weight of roots and shoots was observed at early time points (7, 14 dpi). However, roots of *NahG* transformants were comparatively more sensitive to the root application of SA, which induced a reduction of biomass in mock-inoculated plants at 7 dpi. At 21 dpi, a significant reduction of root and shoot biomass was observed in all *V. longisporum*-inoculated *NahG* transformants. Except for root biomass at 21 dpi without exogenous application of SA, biomass of wild type plants was not significantly reduced by *V. longisporum* inoculation. Correspondingly, significantly higher amounts of fungal DNA were found in the hypocotyls of *NahG* transformants compared to wild type plants (Figure 1A). Exogenous SA treatment did not significantly reduce the amount of fungal DNA (Figure 1B).

### 2.2. Endogenous SA in the Hypocotyl of Wild Type and NahG Transformant Plants

*NahG* transformants were not able to accumulate free SA upon infection with *V. longisporum*, while in wild type plants a significant increase of free SA was observed in *V. longisporum* infected plants, especially two weeks after inoculation (Figure 1C), when *V. longisporum* biomass in hypocotyls significantly increased in *NahG* transformants but not in wild type plants. Exogenous application of SA did not affect the level of free SA in plant tissue (Figure 1D). Free SA showed a slight negative correlation with logarithm of *V. longisporum* DNA (r = −0.24, *p* = 0.09). 

### 2.3. *V. longisporum* Disease Development in Resistant and Susceptible Cultivars

**Figure 2:**
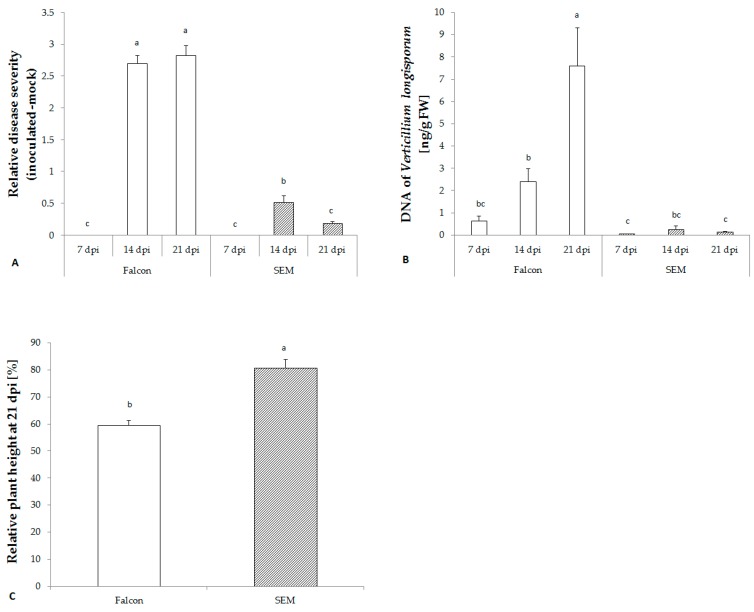
Development of *V. longisporum* in *B. napus* cv. Falcon and SEM determined by disease severity (**A**), fungal biomass in hypocotyls (**B**), and plant height at 21 dpi (**C**). Bars indicate standard errors. Different letters indicate significant differences among the treatments (LSD test, *p* < 0.05). FW, fresh weight.

No visual symptoms were observed during the first week after inoculation in either cultivar. However, two weeks after inoculation with *V. longisporum*, the susceptible cultivar Falcon began to display significantly more severe disease symptoms on leaves and a stronger stunting, while the resistant cultivar only exhibited a slight reduction of plant height (Figure 2A,C). A significant accumulation of *V. longisporum* DNA in the hypocotyl of the susceptible cultivar was detected which was lacking in the resistant cultivar SEM confirming the disease phenotyping data (Figure 2B).

### 2.4. Changes in the SA Biosynthetic Pathway of B. napus Infected with V. longisporum

**Figure 3:**
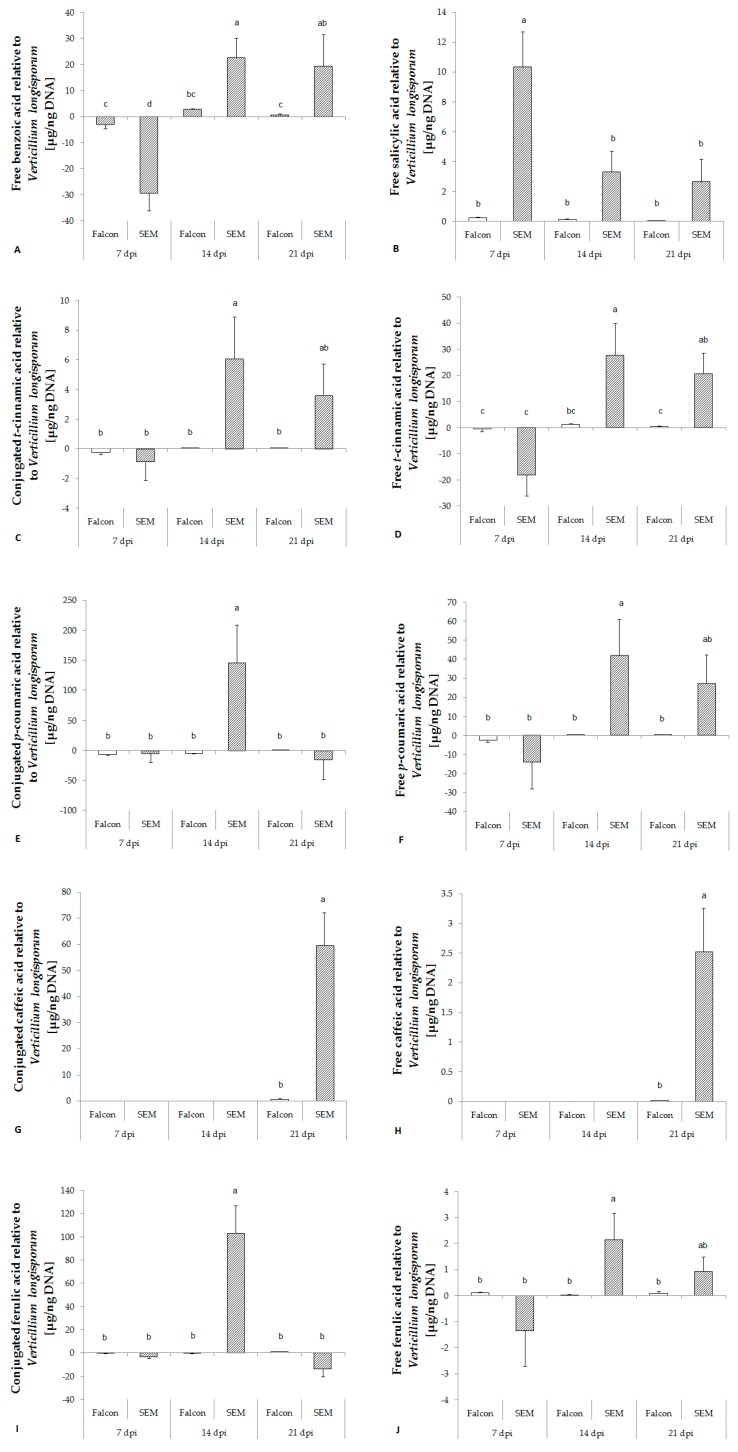
Response of free benzoic acid (**A**), salicylic acid (**B**) and different phenolic acids (**C–L**) in the hypocotyl of *B. napus* cv. Falcon and SEM upon infection of *V. longisporum*. Bars indicate standard errors. Different letters indicate significant differences among the treatments (LSD test, *p* < 0.05). FW, fresh weight; n.s., no significant difference.

**Figure 4:**
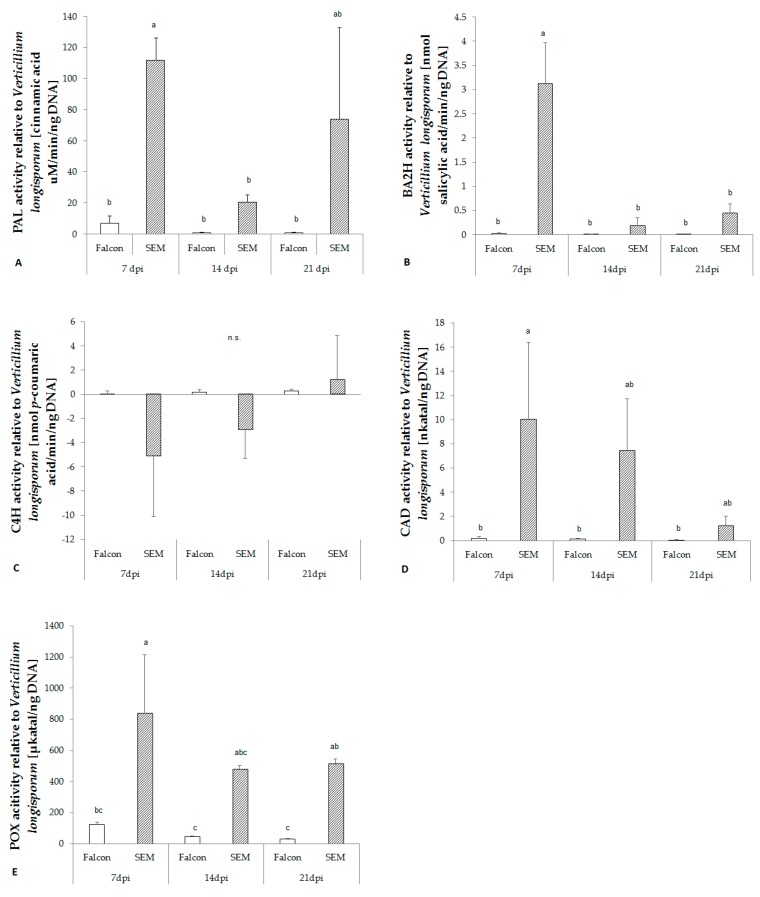
Enzyme activity in the hypocotyl of *B. napus* cv. Falcon and SEM inoculated with *V. longisporum*. (**A**) PAL, phenylalanine ammonia lyase; (**B**) BA2H, benzoic acid 2-hydroxylase; (**C**) C4H, cinnamate 4-hydroxylase; (**D**) CAD, cinnamyl alcohol dehydrogenase; (**E**) POX, peroxidase. Bars indicate standard errors. Different letters indicate significant differences among the treatments (LSD test, *p* < 0.05). n.s. means no significant difference.

**Figure 5:**
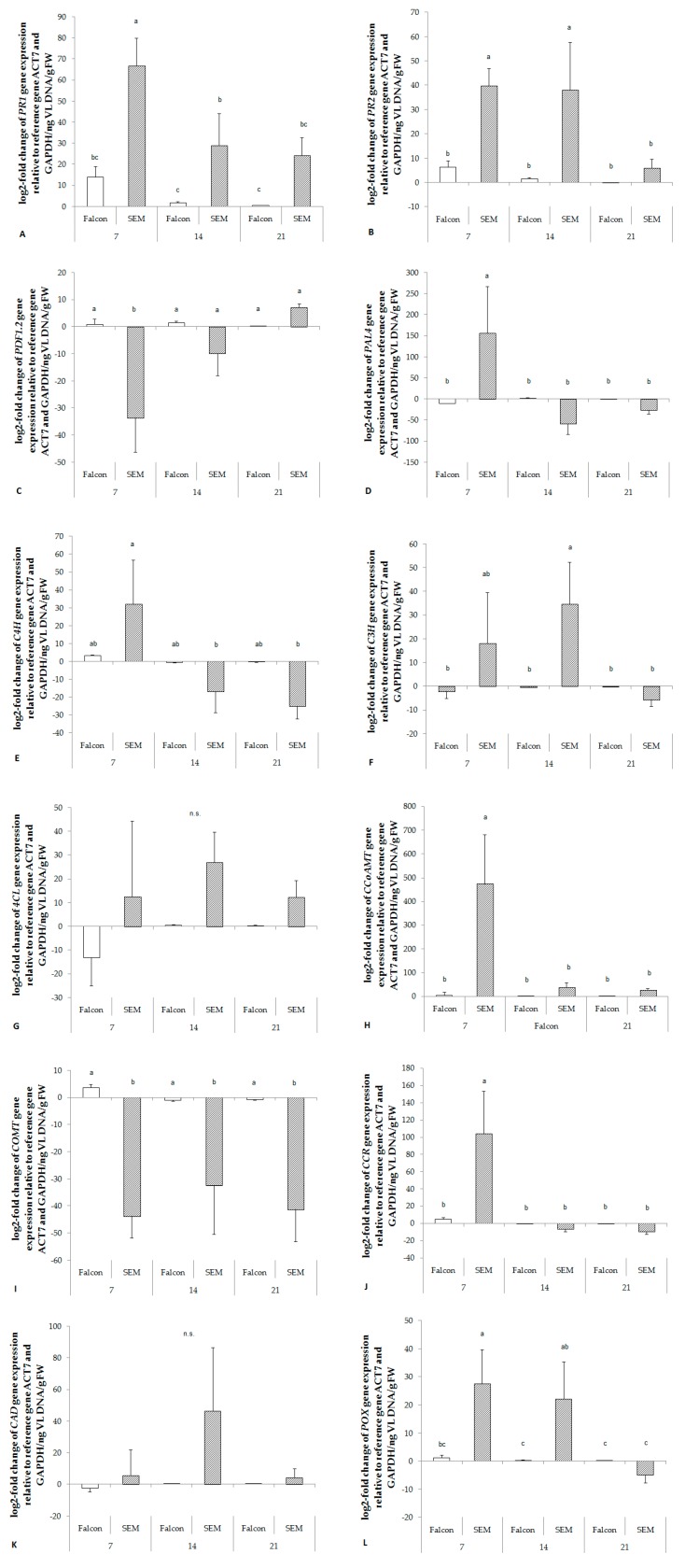
Fold change of salicylic acid mediated resistance marker genes (**A, B**), jasmonic acid mediated resistance marker gene (**C**), and genes of key enzymes involved in lignin synthesis (**D–L**) in the hypocotyl of *B. napus* after infection by *V. longisporum*. The change rates were derived from a comparison of mock-inoculated plants. Target gene expression was normalized to the expression of *ACT7* and *GAPDH*. The log2-fold change was related to the fungal biomass. *PR1*, pathogenesis-related protein 1; *PR2*, pathogenesis-related protein 2; *PDF1.2*, plant defensin 1.2; *PAL4*, phenylalanine ammonia lyase; *C4H*, cinnamate 4-hydroxylase; *C3H*, *p*-coumarate 3-hydroxylase; *4CL*, 4-coumarate:CoA ligase; *CCoAMT*, caffeoyl-CoA 3-O-methyltransferase; *COMT*, catechol-O-methyl transferase; *CCR*, cinnamoyl-CoA reductase; *CAD*, cinnamyl alcohol dehydrogenase; *POX*, peroxidase; *ACT7*, actin 7; *GAPDH*, Glyceraldehyde-3-phosphate dehydrogenase. Bars indicate standard errors. Different letters indicate significant differences among the treatments (LSD test, *p* < 0.05). FW, fresh weight; VL, *V. longisporum*; n.s., no significant difference.

In the absence of disease symptoms of *V. longisporum* infection, free benzoic acid (BA) in the resistant cultivar SEM was strongly used for the production of free SA which was evident by fast increase of free SA at the same time (Figure 3A,B). Similar to free SA, the activity of benzoic acid 2-hydroxylase (BA2H) was transiently enhanced at 7 dpi by infection. In general, responses in both metabolite levels and enzyme activities relative to fungal biomass in the susceptible cultivar Falcon were low (Figure 4B). Besides, a stronger increase in expression of SA mediated marker genes *PR1* and *PR2* relative to the fungal biomass was found in the resistant cultivar (Figure 5A,B), while the jasmonic acid mediated marker gene *PDF1.2* was down-regulated (Figure 5C).

### 2.5. Changes in Phenolic Acid Levels related to Lignin Biosynthesis in Response to V. longisporum Infection

Phenolic acids are formed in the lignin biosynthetic pathway in the following order: *trans*-cinnamic acid (*t*CA), *p*-coumaric acid (*p*CA), caffeic acid (CA), ferulic acid (FA), sinapic acid (SiA). In healthy *B. napus* plants, the contents of all conjugated phenolic acids increased with growth, whereas free phenolic acids were relatively stable in content all over the time of sampling (*data not shown*). In contrast to SA, no significant regulation of phenolic acids was found in either cultivar one week after inoculation with *V. longisporum*. Except CA and free SiA, significant increases of *t*CA, *p*CA, FA and conjugated SiA toward infection of *V. longisporum* were detected in the resistant cultivar at 14 dpi (Figure 3). The amount of CA was under detection limit until 14 dpi, but a clear increase was found in the resistant cultivar at 21 dpi (Figure 3G,H).

### 2.6. Activity of Key Enzymes in Lignin Biosynthetic Pathway

The activity of phenylalanine ammonia lyase (PAL) towards infection of *V. longisporum* was remarkably increased in the resistant cultivar at 7 dpi, while in the susceptible cultivar less activity was found (Figure 4A). Prior to production of phenolic acids, the activity of cinnamyl alcohol dehydrogenase (CAD) and peroxidase (POX) increased in the resistant cultivar at 7 dpi (Figure 4D,E). However, cinnamate 4-hydroxylase (C4H), which catalyzes conversion of *t*CA to *p*CA, did not differ between resistant and susceptible cultivars (Figure 4C).

### 2.7. Regulation of Genes of Key Enzymes involved in Lignin Synthesis

Similar to enzyme activity, expression of almost all genes involved in lignin synthesis (*PAL4*, *C4H*, *C3H*, *CCoAMT*, *CCR*, *CAD* and *POX*) was up-regulated relative to fungal biomass in the resistant cultivar already at 7 dpi. In contrast, *COMT* was strongly down - regulated in the resistant cultivar (Figure 5). No significant responses were found in the expression of *4CL* (Figure 5G). 

### 2.8. Principal Component Analysis

**Figure 6:**
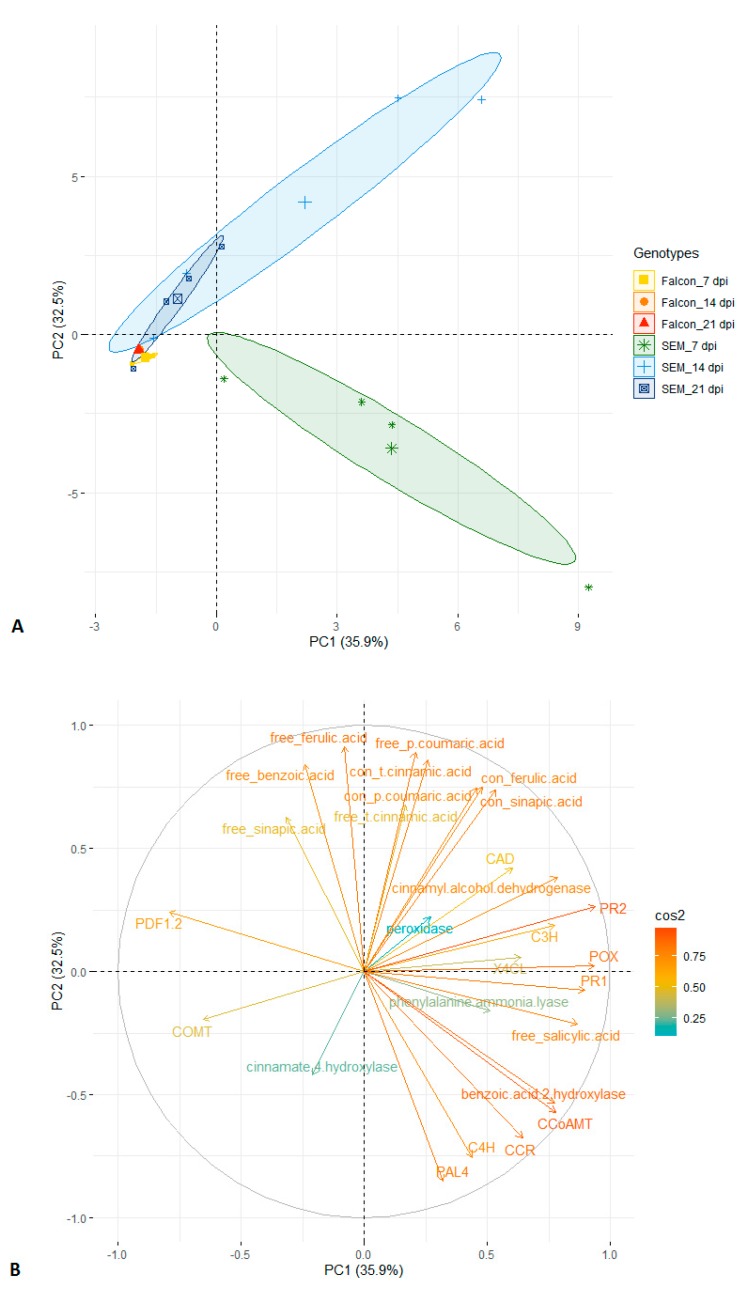
Principal component analysis of twenty-seven traits (metabolites, enzyme activities and gene expression) recorded in two *B. napus* genotypes with *V. longisporum* infection (**A**) and circles of cos^2^ value of the variables (**B**). Labels of traits in capital letters are names of genes. Numbers of clusters were determined by elbow method. PC, principal component; con, conjugated form.

Principal component analysis is a powerful statistical tool for multivariate experiments. Using principal components 1 and 2 based on 27 traits, a clear separation of resistant and susceptible cultivars is possible, with the resistant cultivar at 7 dpi being most distinct cluster (Figure 6A). The distributions of cos^2^ values among principal components indicate that gene expression of *PR1*, *PR2*, *POX* and level of free SA were the strongest contributors to the component 1, while component 2 was mainly determined by the level of free FA, free *p*CA, conjugated *t*CA (Figure 6B). These parameters seem to be the best variables to explain the variation in response of both genotypes to fungal infection. 

## 3. Discussion

### 3.1. SA Plays a Role in Basal Resistance of B. napus to V. longisporum

SA, an important phytohormone involved in disease defense, has been shown to play a role not only in local resistance but also in the activation of systemic acquired resistance (SAR) [10]. Most of the SA synthesized in plants is modified by glucosylation and methylation, which are induced upon pathogen infection [14]. Free SA can be released from this inactive storage form when necessary and maintain SAR over extended periods of time [19]. Methyl SA, a volatile ester, as well as free and conjugated SA in tobacco were induced after infection with avirulent strains of *Pseudomonas syringae* [20]. However, overexpression of glucosyl and methyl transferases in *A. thaliana* suppressed the accumulation of SA and SA-glucoside, and the *AtSGT1* and *OsBSMT1* mutants became more susceptible to disease [21,22]. Like SA and SA-glucoside concentrations in the xylem sap of *B. napus* [15], the concentrations of free SA in wild type plants measured in the present study were strongly modulated by infection of *V. longisporum*. *V. longisporum* penetrates the roots within 60 hpi and starts to colonize the xylem of the shoot three weeks after inoculation [23]. Accordingly, an increase of free SA in the hypocotyl was observed in wild type plants one week after inoculation in the present study. Previous studies showed that the SA-dependent defense pathway was not effective to increase resistance to all pathogens studied [13,14]. SA has been reported to be involved in basal defense and to induce resistance to *Oidium neolycopersici* in tobacco and to *Botrytis cinerea* in tomato; however, SA-deficient *NahG* transformed tobacco and tomato did not enhance the susceptibility to *B. cinerea* and powdery mildew, respectively [13,14]. As described by Johansson et al. [24], no enhanced susceptibility to *V. longisporum* was found in a *Nah*G mutant of *Arabidopsis*. The *Nah*G transformant in the present study showed a remarkable increase in susceptibility to *V. longisporum,* highlighting that SA probably plays an important role in basal defense of *B. napus* against *V. longisporum*. Although no linear correlation exists between SA levels and fungal growth, a threshold level of SA is required for resistance of *B. napus* to *V. longisporum*. 

However, endogenous free SA was not accumulated by exogenous application of 0.5 mM of SA to the roots, which neither had direct negative effects on *V. longisporum* nor was it phytoxic to *B. napus* [25]. In *Arabidopsis*, SA pretreatment did not show any significant alterations in fresh weight loss or symptoms caused by *V. longisporum* [24]. Similarly, in our experiment, no clear reduction in *V. longisporum* biomass and in dry weight losses of roots and stems was observed in SA pretreated *Nah*G transformants and wild type plants. 

### 3.2. Role of SA and Phenolic Acids in Cultivar Resistance of B. napus to V. longisporum

Cultivar-related resistance was associated with a significantly higher increase of SA relative to fungal biomass in the early asymptomatic interaction stage (7 dpi). The resistant cultivar reacted much stronger by shifting more BA to SA. SA can also directly bind to NPR1, the core compound of the SA signaling network, forming a copper-binding transcription-regulator to activate the expression of *PR1* [26]. In contrast to *A. thaliana* [17], *B. napus* showed a strong enhancement of expression of SA-mediated marker genes *PR1* and *PR2* upon *V. longisporum* infection, while JA-dependent *PDF1.2* was relatively stable or even down-regulated. 

Lignin is an aromatic polymer that is deposited during secondary cell wall thickening providing a physical barrier against initial pathogen penetration and colonization. The biosynthesis of SA from phenylalanine and lignin synthesis both depend on PAL activity, which may lead to a competitive relationship between these two synthesis pathways [27]. The phenylalanine route branches into lignin synthesis from *t*CA catalyzed by the enzyme C4H, and the synthesized lignin monomers are transported to the cell wall and polymerized by POX [28]. In *Arabidopsis*, soluble phenylpropanoids were involved in the defense response against *V. longisporum* infection. Such accumulated soluble phenolic compounds may be toxic to pathogens [16]. Maury et al. [29] demonstrated that tobacco compromised in O-methyltransferase activity produced lower amounts of phenolic bacterial virulence gene inducers and thus had smaller tumors caused by *Agrobacterium tumefaciens*. Previous studies showed that both conjugated and free phenolic acids were induced by *V. longisporum* infection, and higher levels were found in hypocotyls of resistant cultivars of *B. napus,* thus indicating a role in cultivar resistance [18]. 

In the present study, production of SA declined in the resistant cultivar starting from 14 dpi, which coincided with a strong increase in levels of phenolic acids. However, enzymes involved in lignin synthesis were already activated at 7 dpi, which occurred alongside with an enhanced expression of the related genes. Our study showed consistently higher activity of POX in the resistant cultivar from 7 to 21 dpi and a strong up-regulation of *POX* until 14 dpi indicating that the resistant cultivar may have accumulated sufficient amounts of lignin by 21 dpi after infection, which is in agreement with previously shown histochemical analyses [18]. 

*V. longisporum* is considered a hemibiotroph, which has a biotrophic life phase in the roots and the xylem and a late necrotrophic phase in the stem parenchyma [30]. As described in previous studies, some pathogen effectors, such as VdIsc1 secreted by *V. dahliae*, can target SA signaling in plants to enhance virulence by preventing SA accumulation [31]. Necrotrophic pathogens, such as *B. cinerea* and *Alternaria solani*, have been reported to enhance the SA signaling pathway in order to antagonize jasmonic acid and to promote disease development in tomato [32]. Accordingly, in the early biotrophic stage of infection, *V. longisporum* may be able to secrete an effector targeting SA synthesis and thus reducing SA levels in susceptible cultivars and allowing higher infection. Since SA is important for basal resistance in oilseed rape, and SA may be induced by infection with biotrophic or hemibiotrophic pathogens [31], avirulent strains of viruses or biotrophic fungi may be efficient biocontrol agents to prevent infection with *V. longisporum* by inducing enhanced levels of SA.

Until present, responses of *B. napus* to *V. longisporum* infection on the metabolomic and transcriptomic level were assessed in relation to plant but not to fungal biomass. This resulted in poor contrasts between responses in resistant and susceptible tissues as their expression was similar related to the same sample volume although responses relative to fungal biomass strongly differed. We believe that the latter is more relevant to accurately describe plant responses to a certain unit of pathogen biomass.

## 4. Conclusions

In summary, a remarkable increase of susceptibility to *V. longisporum* was observed on SA-deficient transgenic *Nah*G oilseed rape indicating that SA plays a role in basal resistance of *B. napus* to *V. longisporum*. In cultivar-related resistance, a stronger increase of SA relative to fungal growth was observed in the resistant cultivar, indicating an important role of elevated SA in defense during early stages of infection, while at later stages, when SA responses do not differ anymore between cultivars, higher levels of phenolic acids are associated with cultivar resistance.

## 5. Materials and Methods

### 5.1. Plant Material and Cultivation

Four cultivars and genotypes, *B. napus* cv. Drakkar, *NahG* transformed Drakkar, Falcon (Norddeutsche Pflanzenzucht Hans-Georg Lembke KG, NPZ, Hohenlieth, Germany), SEM 05-500256 (SEM, Syngenta, Germany) were used in the study. Cultivar Falcon is a susceptible German commercial winter oilseed rape cultivar, and SEM is a winter type breeding line resistant to *V. longisporum* [18]. Seeds of these cultivars were surface sterilized with 70% ethanol for 1 min under constant shaking and subsequently rinsed with sterilized ddH_2_O and pre-germinated in quartz sand. Plants were kept in a climate chamber with a 16 h photoperiod and a temperature of 22 ± 2 °C for 12 days before root inoculation with *V. longisporum*.

### 5.2. Treatments and Experimental Design

To study the basic function of SA and its role in cultivar-related resistance, pot experiments were conducted in a completely randomized block design under climate chamber conditions and repeated twice. The study on the basic function of SA consisted of a combination of three experimental factors, which were genotype (a spring oilseed rape genotype Drakkar and its *NahG* transformant), disease (mock-inoculated and *V. longisporum*-inoculated) and root-dip treatment with SA or water. Treatments were arranged with four biological replicates each composed of 10 plants grown independently in separate pots. The study of cultivar-related resistance consisted of a combination of two experimental factors, which were genotype (two winter oilseed rape genotypes Falcon and SEM) and disease (mock-inoculated and *V. longisporum*-inoculated). Treatments were arranged in a randomized pattern with four biological replicates each composed of 20 plants grown independently in separate pots.

### 5.3. Production of Transgenic *B. napus* Expressing the *NahG* Gene

**Figure 7:**
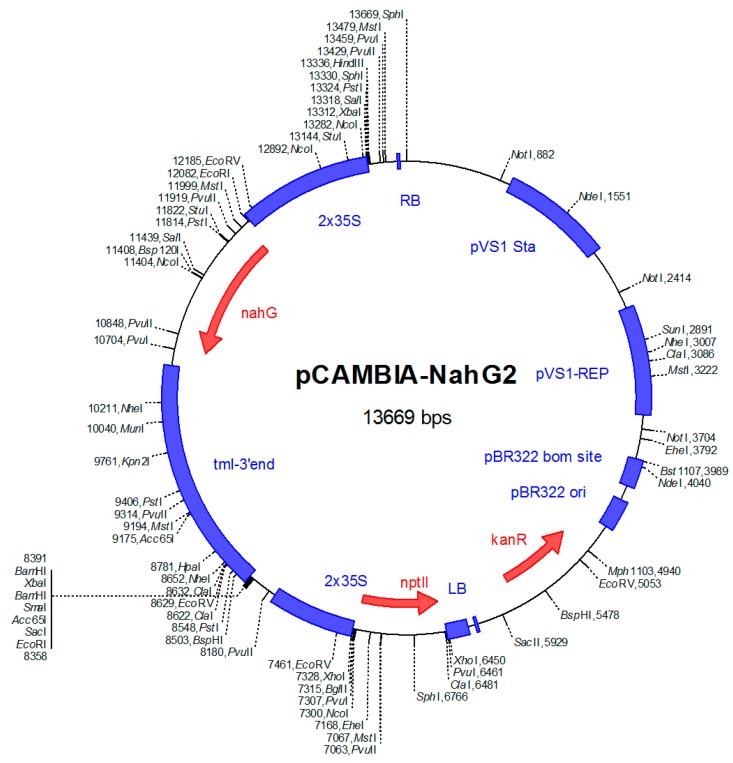
Structure of binary plasmid pCAMBIA2300 containing the *NahG* gene. A fragment of double 35S-*NahG* gene-tml-3´end from pCIB200-*NahG* was inserted into pCAMBIA2300 by using *Xba* I restriction enzyme recognition site. Kanamycin-resistance (*kan*R) marker was the selectable marker used for transformed plant cells. Neomycin phototransferase II (*npt* II) was the selectable marker for plant transformation.

Seeds of transgenic OSR carrying the *NahG* gene were kindly provided by Christian Möllers (Division of Plant Breeding, University of Göttingen). The plasmid used for transformation had been constructed by Corinna Thurow and Christiane Gatz (Institute of Plant Biochemistry, University of Göttingen). Briefly, hypocotyl segments of *B. napus* cv. Drakkar (spring type) were transformed by *Agrobacterium* mediated transformation [33,34]. A binary plasmid, pCAMBIA2300 (Figure 7) containing the *Nah*G gene of *Pseudomonas putida* ND6, which encodes salicylate hydroxylase [35] for degradation of SA to catechol, was used for construction of *Agrobacterium* strain AGLO. A northern blot analysis was performed for expression analysis of transformed plants. Endogenous SA levels in hypocotyl and shoot tissues of transformed plants were measured. One transformant, which had the strongest expression of the *Nah*G gene and low levels of SA, was used for the study.

### 5.4. Exogenous Application of SA

Prior to inoculation with *V. longisporum* by the root-dip method, roots of Drakkar and its *Nah*G transformant were dipped in 0.5 mM of SA or sterile tap water for 24 h. After treatment, plants were rinsed with sterile tap water several times and dried on clean filter papers.

### 5.5. Fungal Culture and Inoculation

*V. longisporum* isolate VL43 obtained from a diseased *B. napus* plant [4,36] was used for inoculations. Conidial suspension kept in 25% glycerol at −80 °C was used to initiate fresh cultures. Fungal cultures were prepared from 400 µl stock conidial suspension added to 250 ml of autoclaved (121 °C, 20 min) potato dextrose broth and then incubated on a rotary shaker at 80 rpm at 22 °C for 10 days. The resulting suspension was filtered through sterile gauze to remove mycelia. 

Twelve-day-old seedlings with cotyledons completely unfolded were inoculated or mock-inoculated using a root-dip method [18]. Plant roots were rinsed with tap water and dipped in a conidial suspension (1 × 10^6^ cfu/ml) or water for 50 min and replanted in pots (7 × 7 × 8 cm) containing a sterile soil-sand mixture (3:1). 

### 5.6. Disease Assessment

According to the assessment key (Table 2) described by Eynck et al. [37], disease severity (DS) was quantified at 7, 14 and 21 dpi. Plant height was measured at 21 dpi. Dry weight of roots and shoots of Drakkar and *NahG* transformants were determined at 7, 14 and 21 dpi.

### 5.7. Extraction and Quantification of Fungal DNA 

Total DNA from hypocotyl samples was extracted using a cetyltrimethylammonium bromide (CTAB) method [38]. About 100 mg of ground fresh plant tissue was suspended in 1 ml CTAB with 2 µl *ß*-mercaptoethanol and 1 µl 1x proteinase K. The extracted DNA was dissolved in 200 µl TE buffer.

A CFX384 real-time PCR detection system (Bio-Rad Laboratories Inc., Kabelsketal, Germany) was used for the amplification and quantification of *V. longisporum* DNA using primers OLG70 (*5′-CAGCGAAACGCGATATGTAG-3′*) and OLG71 (*5′-GGCTTGTAGGGGGTTTAGA-3′*) [39]. The amplification mix consisted of 1× (NH_4_)_2_SO_4_ buffer, 2.5 mM of MgCl_2_, 100 µM of dNTPs, 0.02 U/µl of BioTaq DNA polymerase (Bioline, Luckenwalde, Germany), 0.1x SYBR Green I solution (Invitrogen, Karlsruhe, Germany), 0.3 µM each of primers OLG70 and OLG71 and 1 µl of template DNA and filled up to a total volume of 10 µl with ddH_2_O. PCR conditions were as described in Table 3. PCR for all treatment samples were performed with four biological and three technical replicates and data were analyzed using CFX Manager Software.

### 5.8. Quantification of Endogenous SA

Free SA was extracted from hypocotyl tissue according to a method modified from Kamble et al. [40]. About 200 mg of liquid nitrogen ground fresh hypocotyl samples was suspended in 1.5 ml of acetone, shaked vigorously and centrifuged at 5500 rpm at 4 °C for 45 min. The supernatant was transferred and evaporated in a speed vacuum centrifuge at 30 °C. The residue was dissolved in 1 ml demineralized water, and 1 ml ethyl acetate was added subsequently. The upper phase from the mixture was transferred and evaporated to dryness at 35 °C. The residue was dissolved again in 200 µl of HPLC grade methanol. 

A dilution series of 100 nM to 20 µM of SA dissolved in HPLC grade methanol was used as internal standard. The peak of SA was identified by comparing retention times of samples and standards and confirmed by addition of standard SA to the samples. Before loading into a HPLC vial, all samples or standards were centrifuged at 5000 rpm for 5 min to precipitate unsolvable particles to prevent injection problems. 

A HPLC-fluorescence system consisting of a Varian 410 automatic injector, two Varian 210 pumps with 10 W SS head, a Lichrospher RP-18 column (250 × 4 mm, 5 µm) protected by a Security Guard™ Carbo-H precolumn (4 × 3 mm, 5 µm) kept at 30 °C and a Varian 363 fluorescence detector with excitation wavelength at 315 nm and emission wavelength at 405 nm. Each sample was analyzed for 33 min under a bi-mobile phase with (A) 20 mM sodium acetate, pH 5.0 and (B) methanol with a flow rate of 1 ml/min with the following protocol: initial 10% B for 2 min, linear gradient to 38% B in 13 min, increased to 98% B in 30 s and held for 9 min, equilibrated to initial condition in 30 s and hold for 8 min. The injection volume was 10 µl.

### 5.9. Quantification of Phenolic Acids in Hypocotyls

Phenolic acids were extracted from hypocotyl following published protocols [18,41,42]. About 200 mg of hypocotyl samples ground in liquid nitrogen were suspended in 2 ml of 80% methanol with 0.2 mg/ml of 2,6-di-tert-butyl-4-methylphenol. The mixture was well mixed and sonicated for 10 s. After incubation for 30 min at room temperature, the mixture was centrifuged at 1000 rpm for 10 min. The procedure was repeated twice. 

*Free phenolic acids.* The supernatant was transferred and evaporated in a speed vacuum centrifuge at 30 °C. The residue was dissolved in 1 ml demineralized water, and 0.5 µl of 37% HCl was added to adjust to pH 2–3. Ethyl acetate (1 ml) was used twice for extraction of free phenolic acids from the crude extract. After thoroughly mixing, the upper phase was transferred and evaporated to dryness at 35 °C. The residue was dissolved again in 200 µl of 80% HPLC grade methanol. 

*Methanol-insoluble ester bound phenolic acids.* The pellet after methanol extraction was hydrolyzed with 1 ml of 2 M NaOH at 95 °C for 1 h and mixed four times in between. For acidification of the mixture, 218 µl of 37% HCl was added. Ester bound phenolic acids were extracted twice with 1 ml ethyl acetate. The supernatant was transferred and evaporated to dryness using speed vacuum centrifugation at 35 °C. The residue was dissolved in 200 µl of 80% HPLC grade methanol.

A standard series mix of CA, *p*CA, FA, SiA, *t*CA, BA and SA dissolved in 80% HPLC grade methanol, from 50 ppb to 10 ppm for each compound, was used as internal standards. Peak assignment of each component was made by comparing retention times of samples and standards as well as by comparing the UV absorption spectra (200~500 nm) of analytes to purchased standards. 

The HPLC-fluorescence/DAD system consisted of a JASCO AS-2051 Plus intelligent sampler (4 °C), a DG-2080-54 4-line degasser, an LG-2080-04S quaternary gradient unit, a Kinetex EVO C18 column (250 × 4 mm, 5 µm) protected by a Gemini NX C18 guard column (4 × 3.0 mm, 5 µm) kept in a column oven at 40 °C (CO-2060 Plus Intelligent Column Thermostat), an FP-2020 Plus intelligent fluorescence detector with excitation wavelength at 315 nm and emission wavelength at 405 nm and a MD-2015 Plus multi-wavelength detector measuring over the range of 200~500 nm. Each sample was analyzed for 65 min in a bi-mobile phase run with (A) 0.1% phosphoric acid in water and (B) 0.1% phosphoric acid in acetonitrile with a flow rate of 1 ml/min following this protocol: initial 9% B for 5 min, linear gradient to 32% B in 39 min, increased to 98% B in 30 s and hold for 10 min, equilibrated to initial condition in 30 s and hold for 10 min. The sample injection volume was 10 µl. 

All analytical data from metabolite assays were recorded as µg per g plant FW and related to fungal biomass measured in the plant tissue as ng fungal DNA per g plant FW. 

### 5.10. Enzyme Assays

The hypocotyls were harvested from experimental plants (Falcon and SEM) at 7, 14, 21 dpi. Eight to twelve fresh plant samples were ground in liquid nitrogen as one pooled sample. Four independent samples were taken from each treatment at each time point. Each sample had three technical replicates. 

BA2H was extracted as described by Leon et al. [43]. About 100 mg powder was suspended in 500 µl of extraction buffer (20 mM Hepes, containing 10 mM sorbitol, 1% polyvinylpyrrolidone (PVP), 1 mM phenylmethylsulfonyl fluoride, 12.5 mM *ß*-mercaptoethanol, adjusted with NaOH to pH 7.0). The suspension was mixed, sonicated for 2 min and mixed again before being centrifuged for 10 min at 9,274 rpm and 4 °C. The supernatant was used as the enzyme extract. The reaction mixture (250 µl) contained 20 mM Hepes buffer, pH 7.0, 1 mM NADPH, 1 mM BA and 100 µl enzyme extract, and was incubated for 30 min at 30 °C. To stop the reaction, 125 µl of 15% (w/v) trichloroacetic acid was added. The mixture was centrifuged for 5 min at 10,000 × *g*, the supernatant was extracted twice with 250 µl of ethyl acetate:cyclopentane:isopropanol (100:99:1). The upper organic phase was evaporated to dryness for 1 h at 30 °C and the pellet was resuspended in 200 µl of HPLC grade methanol. The BA2H activity was determined as the rate of conversion of BA to SA. SA was quantified by HPLC as described above.

PAL was extracted according to Kamble et al. [44]. About 200 mg homogenized sample was suspended in 1 ml of 5 mM Tris-HCl buffer, pH 8.3 and centrifuged at 10,000 rpm for 15 min at 4 °C. The supernatant was used as a crude enzyme extract. The activity of PAL was determined as the rate of conversion of L-phenylalanine to *t*CA. The reaction mixture (1 ml) contained 25 mM Tris HCl buffer, pH 8.8, 100 µM L-phenylalanine and 100 μl enzyme extract, and was incubated for 60 min at 30 °C. To terminate the reaction, 400 μl of 2 N HCl was added. For further extraction, 800 μl of toluene was added, and the samples were well mixed and then centrifuged at 1000 rpm for 5 min. The upper toluene layer was measured at 290 nm (HP845 × UV-Visible System) and pure toluene was set as blank. A series of *t*CA with concentrations of 5 μM to 80 μM in toluene was used as standards. Enzyme activity was expressed as change in *t*CA in µM/min/g FW.

C4H was extracted as described previously [45,46,47]. About 100 mg powder was suspended in 600 µl of extraction buffer (100 mM Tris-HCl buffer, pH 7.5 with 15 mM *ß*-mercaptoethanol). The suspension was well mixed, sonicated for 2 min, and mixed again before being centrifuged for 20 min at 15,000 rpm at 4 °C. The supernatant was used as the enzyme extract. The reaction mixture (1.5 ml) contained 100 mM Tris-HCl buffer, pH 7.5, 2 mM NADPH, 1.33 mM *t*CA and 250 µl enzyme extract, and was incubated for 35 min at 30 °C. To stop the reaction, 50 µl of 37% HCl was added and then adjusted to pH 11 with 2 M NaOH. The mixture was extracted twice with 1 ml of diethyl ether. After short centrifugation, the upper organic phase was evaporated to dryness for 65 min at 30 °C and the pellet was resuspended in 500 µl of 1 M NaOH, and measured at 330 nm (µQuant, Bio-Tek). The C4H activity was determined as the rate of conversion of *t*CA to *p*CA.

CAD was extracted as described by Chabannes et al. [48]. About 100 mg powder was suspended on ice in 500 µl of 100 mM Tris-HCl buffer, pH 7.5 with 2% PEG6000, 2% PVP and 5 mM freshly prepared dithiothreitol. The suspension was mixed, sonicated for 2 min, and mixed again before being centrifuged for 10 min at 9,274 rpm at 4 °C. The supernatant was used as the enzyme extract. The reaction mixture (500 µl) contained 100 mM Tris-HCl buffer, pH 8.8, 1 mM NADP, 1 mM coniferyl alcohol and 50 µl enzyme extract, and was incubated for 20 min at 30 °C. The formation of coniferaldehyde was monitored at 400 nm using the molar extinction coefficient of coniferaldehyde (2.1 × 10^4^ M^−1^ cm^−1^), and the activity of CAD was expressed as change in absorbance as nkatal.g^−1^ FW (Sibout et al., 2003; Zhang et al., 2006). 

POX measurement was carried out according to Mandal et al. [49]. About 200 mg of homogenized samples were resuspended in 2 ml of 0.1 M phosphate buffer, pH 7.5, containing 0.5 mM of Na-EDTA and 1% of PVP. The mixture was then centrifuged at 9,000 rpm for 30 min at 4 °C. The supernatant was used as enzyme extract. The activity of POX was determined as the rate of conversion of guaiacol to oxidized (dehydrogenated) guaiacol. The reaction mixture (1 ml) contained 81 mM phosphate buffer, pH 7.0, 4.5 mM guaiacol and 10 μl enzyme extract. After 30 μl of 10 mM, H_2_O_2_ was added to the reaction mixture, the measurement was taken immediately at 470 nm for 7.5 min. The enzyme activity was calculated using the molar extinction coefficient of dehydrogenated guaiacol (26.6 mM^−1^ cm^−1^) and expressed as change in absorbance as μkat/g FW.

Similar to the metabolite assays, enzyme activity data were related to fungal biomass measured in the plant tissue as ng fungal DNA per g plant FW. 

### 5.11. Gene Expression

#### 5.11.1. RNA Extraction and Synthesis of cDNA

About 100 mg of homogenized fresh hypocotyl tissue was suspended in 1 ml TRI-reagent (Invitrogen™, ThermoFisher) and incubated for 15 min at room temperature. Subsequently, 100 µl bromochloropropane was added, and the samples were shaken for 15 s. After incubation for 15 min at room temperature, the mixture was centrifuged for 15 min at 10,159 rpm at 4 °C. For RNA precipitation, 0.5 ml isopropanol was added to the upper aqueous phase (RNA), mixed for 10 s, incubated for 10 min at room temperature, and centrifuged for 10 min at 10,159 rpm at 4 °C. The supernatant was gently discarded, and the pellet was washed with 75% ethanol (freshly prepared in DEPC water). The dried RNA was dissolved in 30 µl DEPC treated ddH_2_O. To check the quality of extracted total RNA, 4.5 µl of RNA was added to a mixture containing 1 × formaldehyde gel-running buffer, 50% formamide, 17.5% formaldehyde, and then incubated for 15 min at 65 °C and immediately chilled on ice to break down the secondary structure. Treated RNA was run on 1.5% agarose gel using TBE buffer.

The samples with good RNA quality were digested again with DNase to remove contaminant genomic DNA before being used for cDNA synthesis. The quality of the digested RNAs was confirmed again on 1.5% agarose gel. The RNA concentration was determined using a microplate spectrophotometer (Epoch, Bio-Tek) at 260 nm and controlled by the ratio OD_260_/OD_280_. For reversing mRNA to cDNA, a First Strand cDNA Synthesis Kit (ThermoFisher) with oligo dT_18_, M-MLV reverse transcriptase and RiboLock RNase inhibitor (1 U/reaction) was used according to the manufacturer’s protocol.

#### 5.11.2. Reverse Transcription Quantitative PCR (RT-qPCR)

Primers for selected genes were constructed by using online primer tools such as Primer3 (Version 4.0), IDT OligoAnalyzer 3.1 with the help of sequence databases (http://www.ncbi.nlm.nih.gov and http://www.brassocadb.org). The sequences (5´ to 3´) of forward (F) and reverse (R) primers of each candidate gene used for RT-qPCR are listed in Table 4. The efficiency of each primer was tested by using standard cDNA copies. RT-qPCR was performed in a CFX384 real-time PCR detection system to determine relative gene expression levels with *actin-7* and *GAPDH* as endogenous reference genes with four independent biological replicates using SYBR Green for staining. PCR conditions were as described in Table 3. A no-template control was included in each experiment. Expression values considering the primer efficiency were normalized to the endogenous reference genes with the formula following Pfaffl [50], and the log2-fold change was related to fungal biomass measured in the plant tissue as ng fungal DNA per g plant FW. 

### 5.12. Statistical Analysis

The experimental data were analyzed as completely randomized designs with four replications using STATISTICA 13.2. All data were normal distributed and analyzed using factorial ANOVA. A multiple comparison was analyzed by Fisher LSD test. To analyze the relationship between disease severity and physiological parameters, Pearson’s linear correlation was performed, and correlation coefficients were calculated. The experimental results were presented as means ± standard error at 5% significance level. A principal component analysis was performed using R package factoextra and FactoMineR.

## Figures and Tables

**Table 1:** Disease severity, plant height and dry weight of mock- and *V. longisporum*-inoculated oilseed rape. Different letters indicate significant differences within rows for each parameter (LSD test, *p* < 0.05). SA, salicylic acid; VL, *V. longisporum* inoculated.

			Water Treatment				0.5 mM SA Root Treatment			
			*NahG*		Wild Type		*NahG*		Wild Type	
			Mock	VL	Mock	VL	Mock	VL	Mock	VL
**Disease severity [1,2,3,4,5,6,7,8,9]**		**7 dpi**	1.1 ± 0.07 **a**	1.3 ± 0.12 **a**	1.0 ± 0.00 **a**	1.1 ± 0.05 **a**	1.2 ± 0.09 **a**	1.3 ± 0.10 **a**	1.0 ± 0.00 **a**	1.0 ± 0.00 **a**
		**14 dpi**	1.2 ± 0.09 **b**	4.3 ± 0.20 **a**	1.1 ± 0.05 **b**	1.2 ± 0.08 **b**	1.2 ± 0.09 **b**	3.2 ± 0.34 **a**	1.0 ± 0.00 **b**	1.3 ± 0.00 **b**
		**21 dpi**	1.4 ± 0.15 **b**	5.5 ± 0.15 **a**	1.2 ± 0.08 **b**	1.6 ± 0.18 **b**	1.4 ± 0.15 **b**	4.9 ± 0.18 **a**	1.2 ± 0.09 **b**	1.6 ± 0.20 **b**
**Plant height [cm]**			22.5 ± 1.3 **c**	6.7 ± 0.4 **d**	34.6 ± 2.8 **a**	23.2 ± 2.0 **c**	24.5 ± 1.5 **bc**	7.4 ± 0.4 **d**	31.5 ± 2.7 **ab**	21.0 ± 1.4 **c**
**Dry weight [mg]**	**7 dpi**	**root**	28 ± 1.1 **a**	30 ± 4.7 **a**	24 ± 3.2 **ab**	31 ± 1.6 **a**	23 ± 1.9 **ab**	17 ± 0.9 **b**	28 ± 1.5 **a**	26 ± 3.8 **ab**
		**shoot**	134 ± 5.1 **ab**	136 ± 18.7 **ab**	136 ± 9.3 **ab**	153 ± 6.9 **a**	115 ± 7.7 **b**	122 ± 15.9 **ab**	134 ± 6.8 **ab**	150 ± 6.3 **ab**
	**14 dpi**	**root**	74 ± 9.5 **a**	64 ± 9.8 **a**	70 ± 16.5 **a**	63 ± 9.4 **a**	61 ± 6.6 **a**	57 ± 7.0 **a**	87 ± 18.6 **a**	63 ± 4.3 **a**
		**shoot**	375 ± 64.2 **a**	309 ± 29.0 **a**	400 ± 51.8 **a**	342 ± 9.4 **a**	296±23.7 **a**	277 ± 25.6 **a**	409 ± 18.6 **a**	339 ± 4.3 **a**
	**21 dpi**	**root**	132 ± 11.1 **b**	70 ± 4.8 **c**	170 ± 8.3 **a**	128 ± 10.9 **b**	116±10.4 **b**	55 ± 5.2 **c**	140 ± 14.9 **ab**	131 ± 10.3 **b**
		**shoot**	662 ± 89.7 **a**	383 ± 80.6 **b**	1144 ± 192.2 **a**	847 ± 192.2 **a**	743±142.4 **a**	380 ± 88.1 **b**	1000 ± 156.4 **a**	684 ± 60.8 **a**

**Table 2:** Assessment key for scoring foliar symptoms induced by *V. longisporum* on *Brassica* species inoculated with the root dip method.

Score	Symptom Development
1	No symptoms
2	Weak symptoms on the oldest leaf (yellowing, black veins)
3	Weak symptoms on the next younger leaves
4	About 50% of the leaves have symptoms
5	More than 50% of the leaves have symptoms
6	Up to 50% of the leaves are dead
7	More than 50% of the leaves are dead
8	Only apex is still alive
9	The plant is dead

**Table 3:** PCR program for quantification of DNA of *V. longisporum* and gene expression assay.

Step	qPCR for Quantification of *V. longisporum*	RT-qPCR for Gene Expression
**Initial Denaturation**	95 °C, 4 min	95 °C, 4 min
**Denaturation**	95 °C, 10 s	95 °C, 10 s
**Annealing**	60 °C, 15 s	64.8 °C, 15 s
**Extension**	72 °C, 15 s	72 °C, 15 s
**Repeat Times**	40 cycles	40 cycles
**Melting Curve Analysis**	55 to 95 °C	60 to 95 °C

**Table 4:** Primers used for reference and candidate genes in the biosynthetic pathway of salicylic acid and lignin.

Gene	NCBI Accession	Primer Sequence		PCR Efficiency [%]	Reference
*4CL*	XM_013895971.1	F	ACGCCGAGATGAAAATCATC	106.2	This study
		R	CCGTCTTTGTCAATGGTCTC		
*ACT7*	NM_001316079.1	F	GCTGACCGTATGAGCAAAG	73.6	Wang et al. 2014
		R	AAGATGGATGGACCCGAC		
*C3H*	XM_013879044.1	F	AGACCAGAGAGGTTCTTGGA	119.4	This study
		R	CGAGTCCAGGGTTTTCAGAC		
*C4H*	XM_013888134.1	F	GTATGTGCCGTTTGGTGTTG	73.8	This study
		R	GGACCTTGGCTTCATTACGA		
*CAD*	XM_013817405.1	F	GGTGGCTTCGCTGACACTAT	70.2	This study
		R	TCACACCCATGTGTCCAACT		
*CCoAMT*	XM_013799238.1	F	TTCAAGGCAGCACACGATAG	127.3	This study
		R	TGCCATACTTGTGGACCGTA		
*CCR*	XM_013836581.1	F	TCCGCTAAGACTTACGCTAATC	74.8	This study
		R	CCTCGTAGACCAGCACATGG		
*COMT*	XM_013793239.1	F	CCGGAAAAAGGGAAAGTGATC	123	This study
		R	TCACATCGAATAAAACCTGACC		
*GAPDH*	XM_013856115.1	F	CGCTTCCTTCAACATCATTCCCA	95.6	Alkooranee et al. 2015
		R	TCAGATTCCTCCTTGATAGCCTT		
*PAL4*	XM_013817346.1	F	GGCACGGACAGTTATGGAGT	96.8	This study
		R	GCCGACTTAGGTAGCGTGAG		
*PDF1.2*	XM_013862352.1	F	ATCACCCTTCTCTTCGCTGCTCTC	109.4	Wu et al. 2016
		R	CATACTCCTGACCATGTCCCACTAG		
*POX*	XM_013786965.1	F	CTCTCTGGGGGTCACACATT	82.3	This study
		R	TGTCGAAAACCGTAGGGGTA		
*PR1*	XM_013877950.1	F	AAAGCTACGCCGACCGACTACGAG	108.8	Alkooranee et al. 2015
		R	CCAGAAAAGTCGGCGCTACTCCA		
*PR2*	AF229403.1	F	GTACGCTCTGTTCAAACCGACCC	109.1	Alkooranee et al. 2015
		R	TTTCCAACGATCCTCCGCCTGA		
